# Supplementary material for: A Bayesian reanalysis of the effects of hydroxychloroquine and azithromycin on viral carriage in patients with COVID-19
Source: PLoS One. 2021 Feb 19;16(2):e0245048. doi: 10.1371/journal.pone.0245048 (PMC7894854; doi:10.1371/journal.pone.0245048)
Supplement: Attachment — Submitted filename: Bayes_HCQ_reviewer_comments (1).pdf [file pone.0245048.s001.pdf]

Reviewer #1: PONE-D-20-11807

This manuscript by Hulme et al. is a data reanalysis study and reply to Gautret et al. 20201. It is entitled: "Reply to Gautret et al. 2002: A Bayesian reanalysis of the effects of hydroxychloroquine and azythromicin on viral carriage in patients with COVID-19".

General context: the original article by Gautret and collaborators came out around mid-March, that is to say very quickly and in the early phase of the COVID-19 epidemic in Europe. It concluded for strong evidence in favour of using hydroxychloroquine to treat patients, and for even stronger evidence for combining hydroxychloroquine with azythromicin. As one of the first clinical study, following in vitro experiments, this paper and its conclusions have been widely used as a support to promote the use of hydroxychloroquine to treat COVID-19 patients. A promotion which, given the urgency of fighting the pandemic, had an influence far beyond the scientific community alone, on public debate, political actions and individual behavior, in several countries. However, this study has been heavily criticized for several aspects of its design which clearly deviates from the good scientific practices prescribed for clinical trials. Most of those criticisms are reminded by the authors here, who also point to an early statistical review of the original paper by Dahly et al.2. The main criticisms in this review include the lack of randomization, the lack of a covariate adjusted analysis (such as age, gender, degree of illness, medical center…), unethical practice by the inappropriate inclusion of control patients who refused the treatment, and the case of patients who were inappropriately dropped from the analysis. As pointed out by the authors of the current manuscript, all of these weaknesses, not to say violations, generate uncertainty as to the findings of the original study, which contrasts with the overestimated positive conclusion of its authors.

Aim of the current study: Hulme and colleagues propose a reanalysis of the above data, in order to draw a more rigorous and precise conclusion, with estimating the associated uncertainty. Therefore, and contrary to the original paper, they use Bayesian A/B tests which enable to quantify the evidence in favour of a given hypothesis. The authors focus on two questions: (i) the main effect of hydroxychloroquine on viral carriage, and (ii) the main effect of combined azythormicin and hydroxychloroquine on viral carriage. The outcome is the fraction of patients in each group (hydroxychloroquine, hydroxychloroquine+azythromicin, comparison group) with a positive test at day 6. Importantly, the main added-value of this study is to quantify the effect of the uncertainty around some key aspects of the data, namely the exclusion of some patients who deteriorated and could not be tested at day 6, and missing tests for some patients in the comparison group. These missing data were discarded in the original study, despite the fact that their influence could be significant given the small samples in each group. In the current study, the authors consider different scenarios with these data, namely conservative ones. They also perform more appropriate tests given the questions of interest. They conclude for substantially weaker evidence than initially reported.
Main comments: This paper is important, timely and fairly well written. It brings up an important twofold message to be reminded: (i) despite the emergency of the situation, all efforts should be made to acquire relevant data in order to be able to draw any reliable conclusion; (ii) given imperfect data, the most appropriate and careful statistical analysis should be performed to draw fair conclusions.
The current paper could be better organized in order to be clearer and more straightforward, focusing on its main message. I recommend the authors:

• To introduce the data early in the paper (groups, sample sizes, specify to which group the untested patients and the ones who deteriorated belonged to, …). Hence Figure 4 should become figure 1.

We agree that this should be moved. Note that we now call figure 4, table 1, and it is now above the figures.

• To shorten the abstract and make it more impactful.

We have done this, removing ~200 words.

• To move all the information in the Results section that pertain to a methodological description, to the Methods section.

We don't view these details as methods descriptions, more as modelling descriptions that are essential to understanding and interpreting the results. We would prefer general method details (that are not specific to the results) to go in the methods, and the modelling details (that are specific to each result) to go in the results section. This is mainly to avoid the fragmentation of the models from the results, making it harder for the reader to remember what modelling details apply to which result.

Regarding the statistical approach that is at the heart of this study, I have the following questions:

• The authors of the original paper could argue that what motivated them to use the hydroxychloroquine is a bundle of objective arguments (the known effect of this molecule, the outcome of recent in vitro tests…). Hence a more appropriate test may only consider two hypothesis, H0 and H+, with 0.5 prior probability for each of them. I recommend the authors to discuss this point of view and eventually report the outcome of the tests based on such prior hypothesis.

We argue that it is important when testing a drug of unknown effects that the possibility of harm is formally incorporated into the statistical modelling. It should be pointed out that the main emphasis of the analyses is on the evidence provided by the different models, expressed as Bayes Factors (e.g. the main results in the abstract, and in the summary in figure 3). The Bayes factors remain unchanged by changing the prior model probabilities.

Whilst it is true that the posterior model probabilities change when changing prior model probabilities, we would rather not duplicate all these results with an extra version. There are already many versions of analyses, to add further ones we think would add complexity without adding much insight.

We do however agree that the choices we made should be justified, and discussed alongside their alternatives. We thus added a new section to the discussion as follows:

Negative effect hypotheses. *In this paper we assigned prior probabilities of 0.5 to H_0 and 0.25 each to H_+ and H_- We assign probability mass to both the negative and positive hypotheses because when testing a drug it is important to know whether it negatively or positively impacts clinical outcomes. A different choice is to consider only H_0 and H_+. With prior probability of 0.5 for both hypotheses, this would have resulted in higher posterior probabilities for H_+ than those reported here. However, these different priors on the hypotheses would not change any of the Bayes factors, because such priors quantify relative plausibility whereas the Bayes factor quantifies relative predictive performance, that is, the extent to which the data warrant a reallocation of plausibility from prior to posterior. This is one of the reasons we have emphasised the interpretation of the Bayes factor rather than the posterior probabilities of the hypotheses.*

• The authors perform several tests on the same data. To what extend the Bayesian approach they used should consider correcting for multiple testing? (see e.g. 3).

This is an interesting question. There are two sources for the multiplicity of tests. The first source is the different ways of categorizing the patients, pertaining to different assumptions about the data. As such it is the data that is different not the modelling. Because of this, the models are applied to different counterfactual versions of the data, and thus these should not be considered competing models. As such there is no multiple testing problem. The second source of multiplicity of testing comes from the fact that we test different models for a given dataset, e.g. H+ H- and H0. Here the multiple testing is accounted for by the prior model probabilities, and this is already incorporated in the analyses. The more models, the less probability mass assigned to each model.

• It could be useful to also report the exceedance probability for H+, a quantity that directly pertain to the question of interest, that is how more probable is my hypothesis of interest compared to alternative ones.

Exceedance probabilities are not commonly computed for the type of questions we are asking in this paper. In the domain of neuroimaging, where they are more prevalent (e.g. Stephan et al 2009 https://www.ncbi.nlm.nih.gov/pmc/articles/PMC2703732/),

exceedance probabilities encode a posterior belief that a particular model k is more likely than any of other model (of K models tested), given the data. If there are two models, then the exceedance probability for model k, can be calculated by computing the integral (0.5,1) of the probability distribution of model probabilities for model k. However, such exceedance probabilities are typically computed when you have a distribution of model probabilities for a given model, such as when model probabilities are separately assigned to each subject of a group. Then it becomes a relevant question, what is the probability that a particular model probability will exceed all others in these subjects. However, in the context of our paper, we do not have model probabilities assigned to each subject, we just have one model probability assigned to each model for the whole group. If there is no distribution of model probabilities, then it doesn't make sense to ask what is the probability of the model probability exceeding all others. It either does or it doesn't.

• In the discussion, the authors develop an interesting explanation around the notion and impact of an underpowered study. For educational reason at least, it would be interesting to explain the influence of the number of samples here and how the Bayesian approach accounts for it compared to a classical, frequentists approach as used in the original paper.

We now added this to the discussion:

Bayesian inference framework is a natural way to incorporate sample size in the analysis. Small sample sizes will lead to more variance and hence less certainty on the estimated model parameters, and as such typically do not provide compelling evidence; as sample size increases, the evidence will generally become stronger, either in favor of H0 (when there is no discernable effect in the data) or in favor of H1 (when the data do show an effect). Thus, as sample size grows the evidence typically grows as well, in a smooth fashion. This continual monitoring of evidence is not easily available in a frequentist framework that involves all-or-none decisions (but see Wald, 1947).

Minor remarks: I noticed the following typos
- In Methods / Data / (2): "We exclude both the patient WHO …"

Corrected.

- In Results / Main effect of HCQ… / 3rd paragraph: "…untested patients were assumed NEGATIVE"

Well spotted.

Conclusion: I do recommend this paper to be published in PLoS ONE, after the authors would have addressed the above comments.

Thank you for your constructive suggestions for improvement and a careful reading of our paper.

References

1. Gautret, P. et al. Hydroxychloroquine and azithromycin as a treatment of COVID-19: results of an open-label non-randomized clinical trial. International Journal of Antimicrobial Agents 105949 (2020) doi:10.1016/j.ijantimicag.2020.105949.
2. Dahly, D., Gates, S. & Morris, T. Statistical review of Hydroxychloroquine and azithromycin as a treatment of COVID-19: results of an open-label non-randomized clinical trial. https://zenodo.org/record/3724167 (2020) doi:10.5281/ZENODO.3724167.
3. Sjölander, A. & Vansteelandt, S. Frequentist versus Bayesian approaches to multiple testing. Eur J Epidemiol 34, 809–821 (2019).

Reviewer #2:

This paper provides a careful re-analysis of Gautret et al. data using Bayesian statistics that these authors presumably do not know (or understand if I may say). The analysis sounds solid and conclusions are fairly presented.
I appreciate the need for the re-analysis of the data of Gautret et al. but it is unclear what the scientific message of the paper is.

The main scientific message is that the more reasonable assumptions you apply to the data the less convincing the evidence for the claims of the original paper. We have shortened the abstract to make this clearer. The last sentences now read:

We found that the statistical evidence was highly sensitive to these data variants. Statistical evidence for the positive effect model ranged from strong for the original data (BF_{+0} ~11), to moderate when including patients who deteriorated (BF_{+0} ~4.35), to anecdotal when excluding untested patients (BF_{+0} ~2), and to anecdotal negative evidence if untested patients were assumed positive (BF_{+0} ~0.6). The fact that the patient inclusions and exclusions are not well justified nor adequately reported raises substantial uncertainty about the interpretation of the evidence obtained from the original paper.

Given that the Gautret et al. study is nearly outdated, and has already been largely commented, the present report in its current form may be suboptimal.

We agree that the time window for the paper has largely passed. We do believe that it is important that this becomes part of the historical and scientific record. Beyond this it can illustrate what Bayesian analyses can offer when questioning the inclusion assumptions of a clinical trial. We think that the edits we have made to this paper in the process of replying to both reviewers have enhanced its educational merit.

My recommendation for the present report to strengthen its scientific message for students and non-expert readers in statistics would be to also provide a clear message on the added-value of Bayesian statistics, for example in an introduction that does not exist yet and that could be short but written in a didactic way. This would provide a theoretical context that may be beneficial to everyone.

In line with this suggestion, we have added the following brief introduction:

In March 2020, at the beginning of the corona virus pandemic, Gautret and colleagues reported the results of a non-randomised open-label case series which examined the effects of HCQ and azithromycin (AZ) on viral load in the upper respiratory tract of SARS-CoV-2 patients. The authors reported that HCQ had virus reducing effects, and that dual treatment of both HCQ and AZ further enhanced virus reduction. At the time, these data triggered urgent speculation whether these drugs should be considered as candidates for the treatment of severe COVID-19. However, questions were quickly raised regarding the study's data integrity, statistical analyses, and experimental design. In light of these questions, we reanalysed the original data by performing a multiverse analysis in which we systematically varied assumptions regarding the inclusion of patients in the analysis, and their clinical status. The Bayesian methods we apply have several advantages in this context. Firstly, in contrast to the frequentist methods applied in the original paper, Bayesian statistics allows for a direct evaluation of the evidence for and against competing hypotheses. In other words, we can go beyond accepting or rejecting the null hypothesis of no treatment effect, by assessing how strong is the evidence provided by the data, for one hypothesis over and another. Secondly, by performing a multiverse analysis in which the data depends on the inclusion assumptions made, we can explore how the strength of evidence for the clinical efficacy of the HCQ treatments depends on which subjects were included or excluded, and what their clinical status was.

Thank you for your constructive suggestions and a careful reading of our paper.
